# Supplementary material for: Duplicated antibiotic resistance genes reveal ongoing selection and horizontal gene transfer in bacteria
Source: Nat Commun. 2024 Feb 16;15:1449. doi: 10.1038/s41467-024-45638-9 (PMC10873360; doi:10.1038/s41467-024-45638-9)
Supplement: Supplementary file 1 — Supplementary Information [file 41467_2024_45638_MOESM1_ESM.pdf]

Supplementary Information for

Duplicated antibiotic resistance genes reveal ongoing selection and horizontal gene transfer in bacteria

Rohan Maddamsetti[1,2], Yi Yao[1,2], Teng Wang[1,2], Junheng Gao[3], Vincent T. Huang[1,2], Grayson S. Hamrick[1,2,4], Hye-In Son[1,2], Lingchong You[1,2,4,5*]

[1]Center for Quantitative Biodesign, Duke University, Durham, NC, USA

[2]Department of Biomedical Engineering, Duke University, Durham, NC, USA.

[3]Department of Biostatistics and Bioinformatics, Duke University School of Medicine, Durham, NC, USA.

[4]Center for Biomolecular and Tissue Engineering, Duke University, Durham, NC, USA

[5]Department of Molecular Genetics and Microbiology, Duke University School of Medicine, Durham, NC, USA

* Email for correspondence: you@duke.edu*

Supplementary Table 1. Proportion of isolates containing duplicated antibiotic resistance genes (ARGs).

Annotation	Isolates	Isolates with Duplicated ARGs	Proportion of isolates with Duplicated ARGs	95% confidence interval
Humans	7777	1104	0.1420	[0.1342, 0.1497]
Plants & Animals	3123	61	0.0195	[0.0147, 0.0244]
Earth	2495	38	0.0152	[0.0104, 0.0200]
Livestock	2025	272	0.1343	[0.1195, 0.1492]
Water	1224	31	0.0253	[0.0165, 0.0341]
Human-impacted	1191	88	0.0739	[0.0590, 0.0887]
Food	1103	46	0.0417	[0.0299, 0.0535]

Supplementary Table 2. Proportion of isolates containing single-copy antibiotic resistance genes (ARGs).

Annotation	Isolates	Isolates with single-copy ARGs	Proportion of isolates with single-copy ARGs	95% confidence interval
Humans	7777	7742	0.9955	[0.9940, 0.9970]
Plants & Animals	3123	2983	0.9552	[0.9479, 0.9624]
Earth	2495	2489	0.9976	[0.9957, 0.9995]
Livestock	2025	1949	0.9625	[0.9542, 0.9707]
Water	1224	1218	0.9951	[0.9912, 0.9990]
Human-impacted	1191	1187	0.9966	[0.9934, 0.9999]
Food	1103	1103	1.000	[1.0000, 1.0000]

Supplementary Table 3. Proportion of isolates containing duplicated genes.

Annotation	Isolates	Isolates with duplicated genes	Proportion of isolates with duplicated genes	95% confidence interval
Humans	7777	7249	0.9321	[0.9265, 0.9377]
Plants & Animals	3123	2659	0.8514	[0.8389, 0.8639]
Earth	2495	2246	0.9002	[0.8884, 0.9120]
Livestock	2025	1795	0.8864	[0.8726, 0.9002]
Water	1224	1100	0.8987	[0.8818, 0.9156]
Human-impacted	1191	1129	0.9479	[0.9353, 0.9606]
Food	1103	1001	0.9075	[0.8904, 0.9246]

Supplementary Table 4. Duplicated ARGs on chromosomes and plasmids.

Annotation	Chromosomal duplicated genes	Plasmid duplicated genes	Chromosomal duplicated ARGs	Plasmid duplicated ARGs
Humans	406,232	47,554	2,392	2,825
Livestock	96,593	10,728	553	814
Human-impacted	49,951	12,330	136	284
Plants & Animals	147,270	30,317	103	136
Food	37,409	6,727	68	101
Water	37,441	4,370	42	83
Earth	78,806	12,200	66	46

Supplementary Table 5. Single-copy ARGs on chromosomes and plasmids.

Annotation	Chromosomal single-copy genes	Plasmid single-copy genes	Chromosomal single-copy ARGs	Plasmid single-copy ARGs
Humans	26,809,779	571,349	78,328	14,708
Livestock	6,158,097	144,296	20,121	3,808
Plants & Animals	10,630,513	613,492	25,991	1,328
Human-impacted	4,695,471	182,323	13,353	1,308
Food	3,436,339	75,345	11,536	1,016
Earth	11,193,086	306,348	30,732	626
Water	4,344,815	97,867	9,076	521

Supplementary Figure 1. Laboratory evolution with *E. coli* K-12 MG1655 demonstrate that antibiotic selection is sufficient to drive the rapid evolution of antibiotic resistance through the duplication of antibiotic resistance genes. Eight populations (two replicates per treatment) were evolved in LB supplemented with one of four antibiotics (2000 µg/mL carbenicillin, 70 µg/mL chloramphenicol, 250 µg/mL kanamycin, or 250 µg/mL spectinomycin). Each panel shows a result generated by Illumina sequencing of ancestral clones and whole-population samples isolated after one day of antibiotic selection.

A. One day of antibiotic selection was sufficient to drive an increase in ARG copy number in all treatments. ARG copy number in the ancestral clones is shown at the pre-treatment Day 0 timepoint for comparison.

B. Genome sequencing reveals targets of positive selection after 1 day of growth under antibiotic treatment. All 8 populations show duplicated antibiotic resistance genes, driven by chromosomal transpositions of the Tn5 mini-transposons (miniTn5). Transposons in the RM7-115-32 population show an increase in copy number in panel A, but are not shown in panel B, because these mobilized into multi-copy 23S ribosomal RNA genes, and their precise chromosomal location is ambiguous.

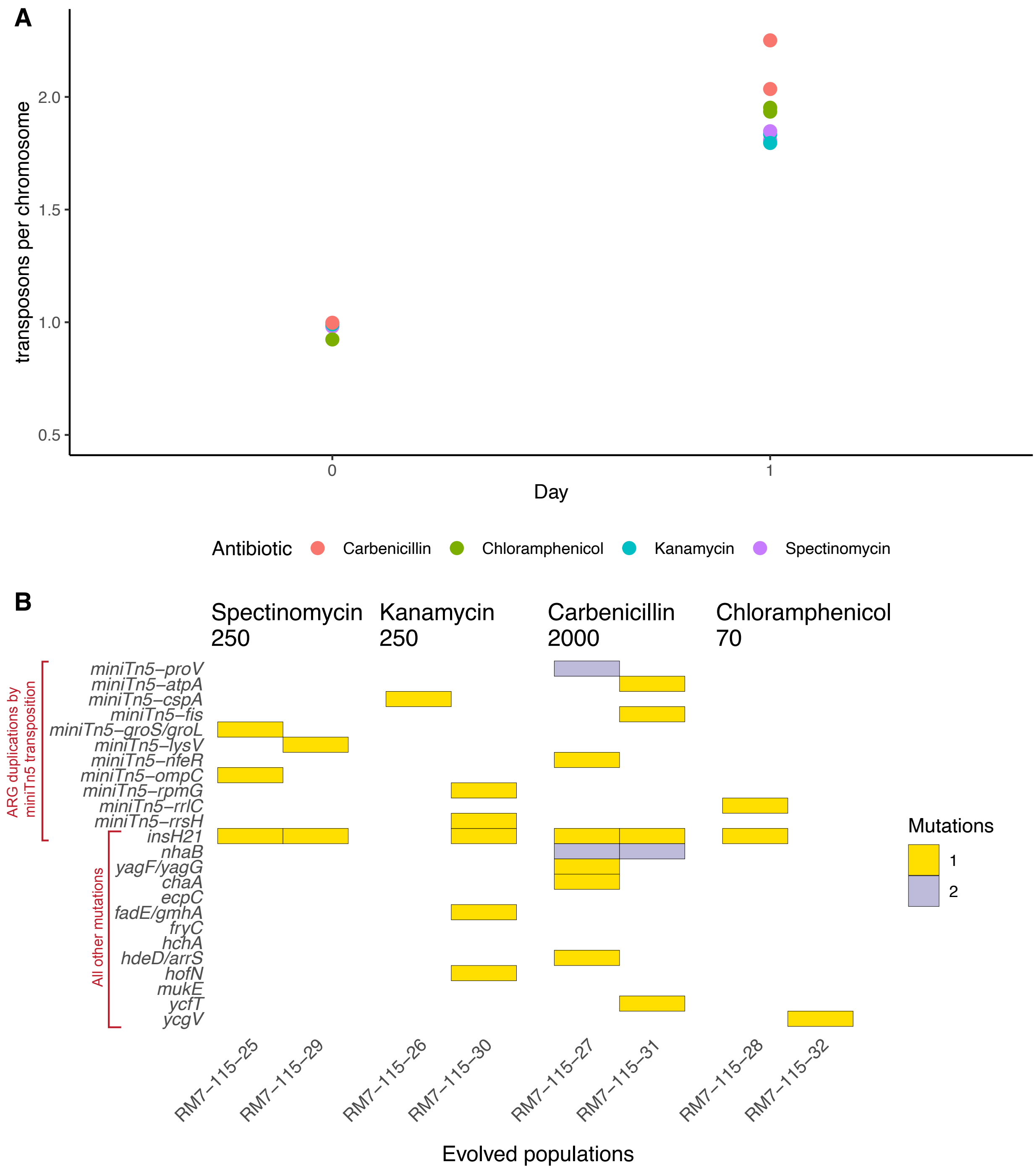
A
transposons per chromosome
2.0
1.5
1.0
0.5
0
1
Day
Antibiotic
Carbenicillin
Chloramphenicol
Kanamycin
Spectinomycin
B
Spectinomycin 250
Kanamycin 250
Carbenicillin 2000
Chloramphenicol 70
ARG duplications by miniTn5 transposition
All other mutations
miniTn5–proV
miniTn5–atpA
miniTn5–cspA
miniTn5–fis
miniTn5–groS/groL
miniTn5–lysV
miniTn5–nfeR
miniTn5–ompC
miniTn5–rpmG
miniTn5–rrlC
miniTn5–rrsH
insH21
nhaB
yagF/yagG
chaA
ecpC
fadE/gmhA
fryC
hchA
hdeD/arrS
hofN
mukE
ycfT
ycgV
RM7–115–25
RM7–115–29
RM7–115–26
RM7–115–30
RM7–115–27
RM7–115–31
RM7–115–28
RM7–115–32
Evolved populations
Mutations
1
2

Supplementary Figure 2. Proportions of isolates with duplicated ARGs, across 12 chemical classes of antibiotics.

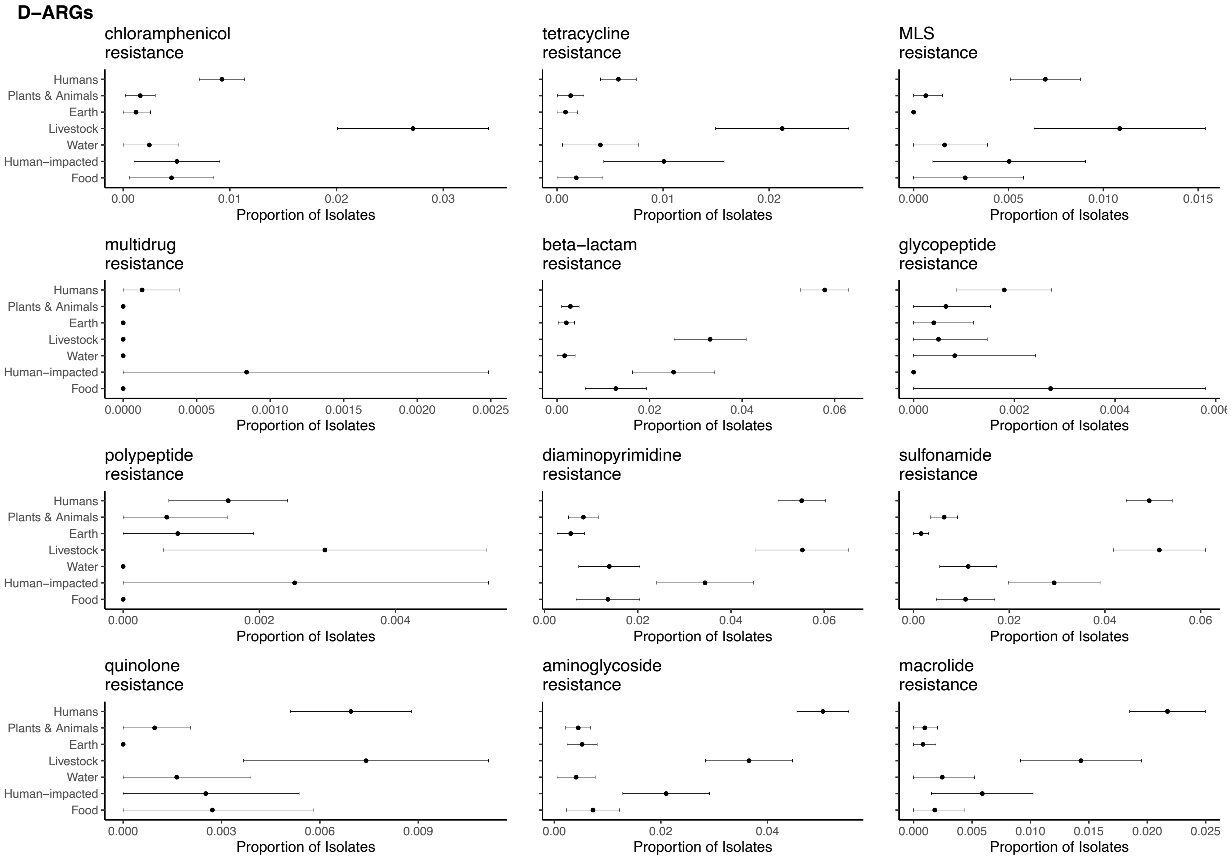

Supplementary Figure 3. D-ARGs are enriched on the chromosomes and plasmids of bacteria isolated from humans and livestock. Error bars are 95% binomial proportion confidence intervals, calculated using the formula $p \pm Z_{\alpha/2}\sqrt{(\frac{p(1-p)}{n})}$, where *p* is the proportion, *n* is the sample size, and $Z_{\alpha/2}$ = 1.96.

A. Bacterial isolates with D-ARGs on chromosomes are specifically associated with humans and livestock.

B. Bacterial isolates with D-ARGs on plasmids are specifically associated with humans and livestock.

C. Most isolates contain at least one single-copy ARG on their chromosome.

D. Bacterial isolates with S-ARGs on plasmids are specifically associated with humans, livestock, and human-impacted environments.

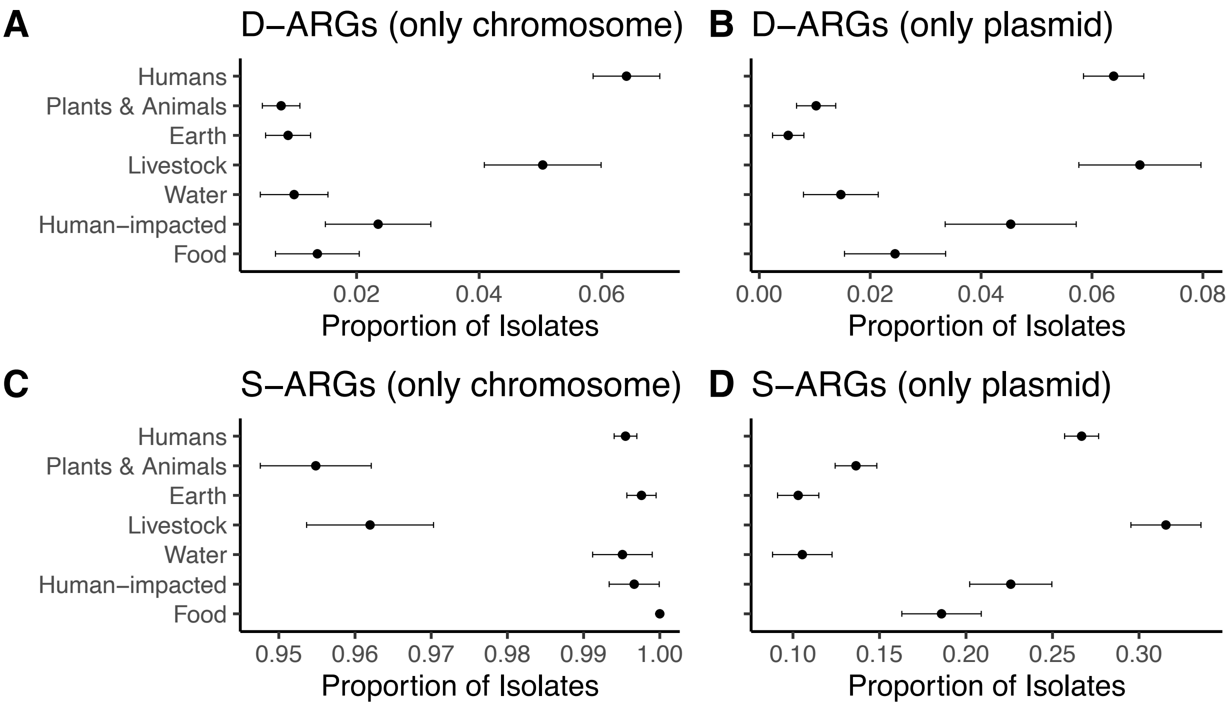

Supplementary Figure 4. Bacteria isolated from humans and livestock are more likely to have duplicated antibiotic resistance genes (D-ARGs) compared to bacteria isolated from other environments, even after taxonomic down-sampling. Error bars are 95% binomial proportion confidence intervals, calculated using the formula $p \pm Z_{\alpha/2}\sqrt(\frac{p(1-p)}{n})$, where *p* is the proportion, *n* is the sample size, and $Z_{\alpha/2}$ = 1.96. A) The number of D-ARGs per bacterial genus positively correlates with the number of isolates per genus. The most common genera in the data are shown in red. B) Removing the genera colored in red in panel A does not affect the enrichment of D-ARGs in livestock (5,638 isolates were removed, leaving 13,300 isolates). C) The same trend is seen, when just examining the genera colored in red in panel A. D) The enrichment of D-ARGs in humans and livestock is maintained, after downsampling the isolates by Mash distance > 0.005. E) The enrichment of D-ARGs in humans and livestock is maintained, after downsampling the data to one genome per species.

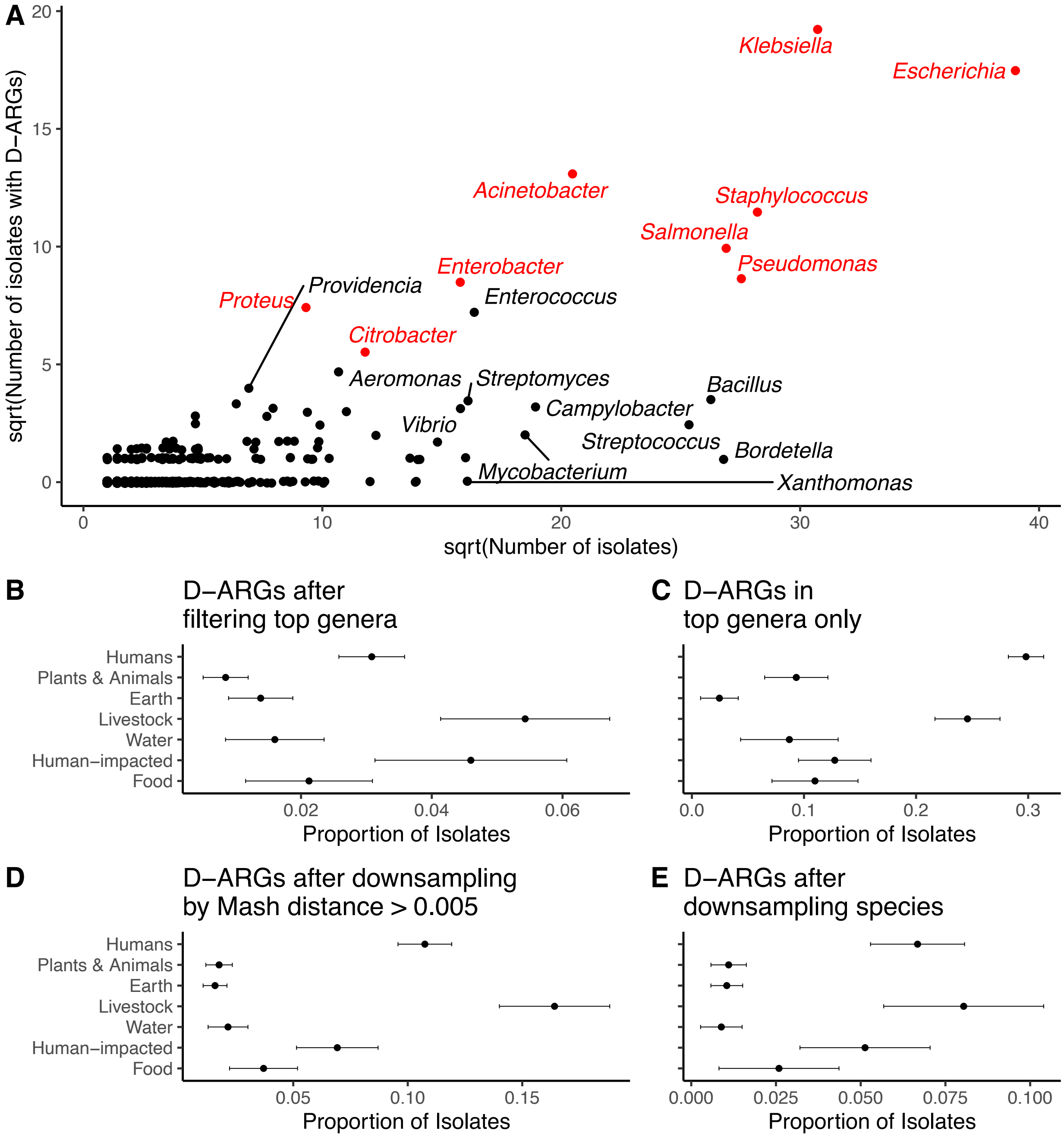
A
sqrt(Number of isolates with D−ARGs)
sqrt(Number of isolates)
0
5
10
15
20
0
10
20
30
40
Klebsiella
Escherichia
Acinetobacter
Staphylococcus
Salmonella
Pseudomonas
Enterobacter
Providencia
Proteus
Enterococcus
Citrobacter
Aeromonas
Streptomyces
Bacillus
Campylobacter
Vibrio
Streptococcus
Bordetella
Mycobacterium
Xanthomonas
B
D−ARGs after filtering top genera
Humans
Plants & Animals
Earth
Livestock
Water
Human−impacted
Food
0.02
0.04
0.06
Proportion of Isolates
C
D−ARGs in top genera only
0.0
0.1
0.2
0.3
Proportion of Isolates
D
D−ARGs after downsampling by Mash distance > 0.005
Humans
Plants & Animals
Earth
Livestock
Water
Human−impacted
Food
0.05
0.10
0.15
Proportion of Isolates
E
D−ARGs after downsampling species
0.000
0.025
0.050
0.075
0.100
Proportion of Isolates

Supplementary Figure 5. Proportions of isolates with single-copy ARGs, across 12 chemical classes of antibiotics.

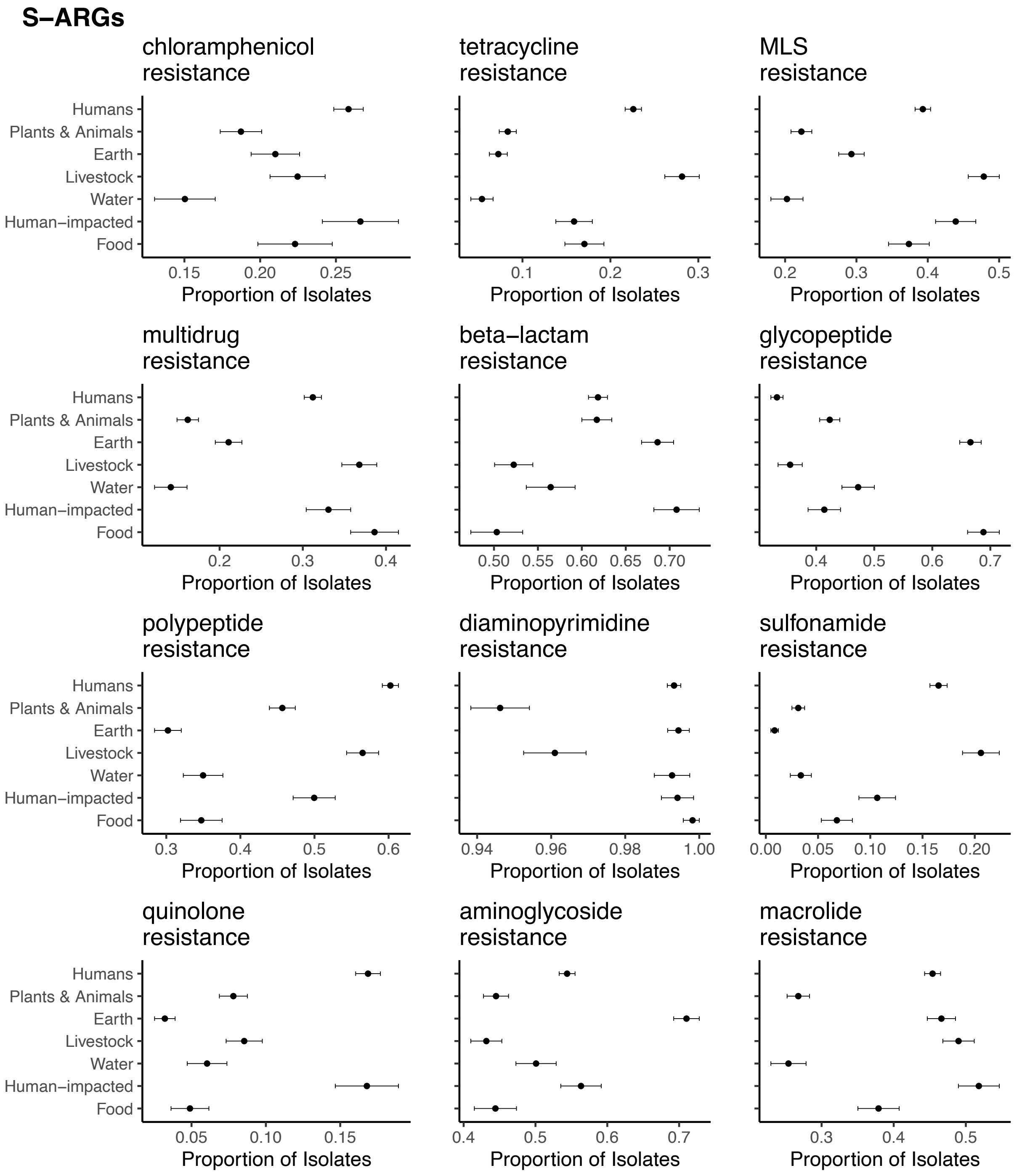

Supplementary Figure 6. Distribution of duplicated ARGs in 12 clinical extended-spectrum beta-lactam (ESBL) resistant *E. coli* isolates from Duke Hospital. Long-read genome assemblies were downloaded from NCBI BioProject PRJNA290784. A) The number of duplicated genes in the 12 isolates is highly variable. 6 out of the 12 isolates harbor duplicated antibiotic resistance genes. B) All 12 isolates have similar numbers and fractions of single-copy genes encoding mobile genetic element functions and antibiotic resistance.

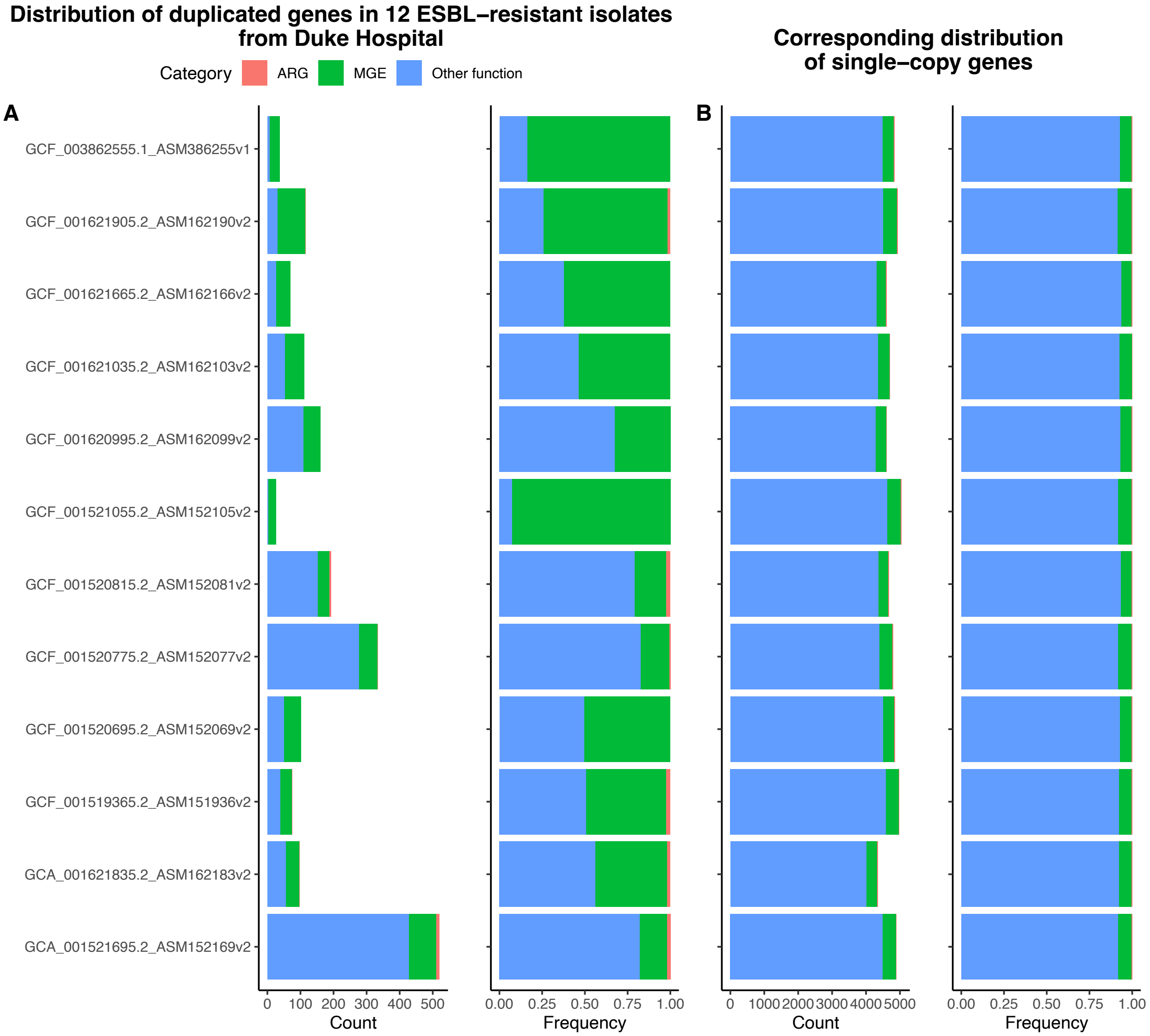

Supplementary Figure 7. Distribution of duplicated ARGs in 46 clinical antibiotic resistant isolates sequenced as part of the BARNARDS study. Long-read genome assemblies were downloaded from NCBI BioProject PRJNA767644. A) The number of duplicated genes in the 46 isolates is highly variable. 23 out of the 46 isolates harbor duplicated antibiotic resistance genes. B) All 46 isolates have similar numbers and fractions of single-copy genes encoding mobile genetic element functions and antibiotic resistance.

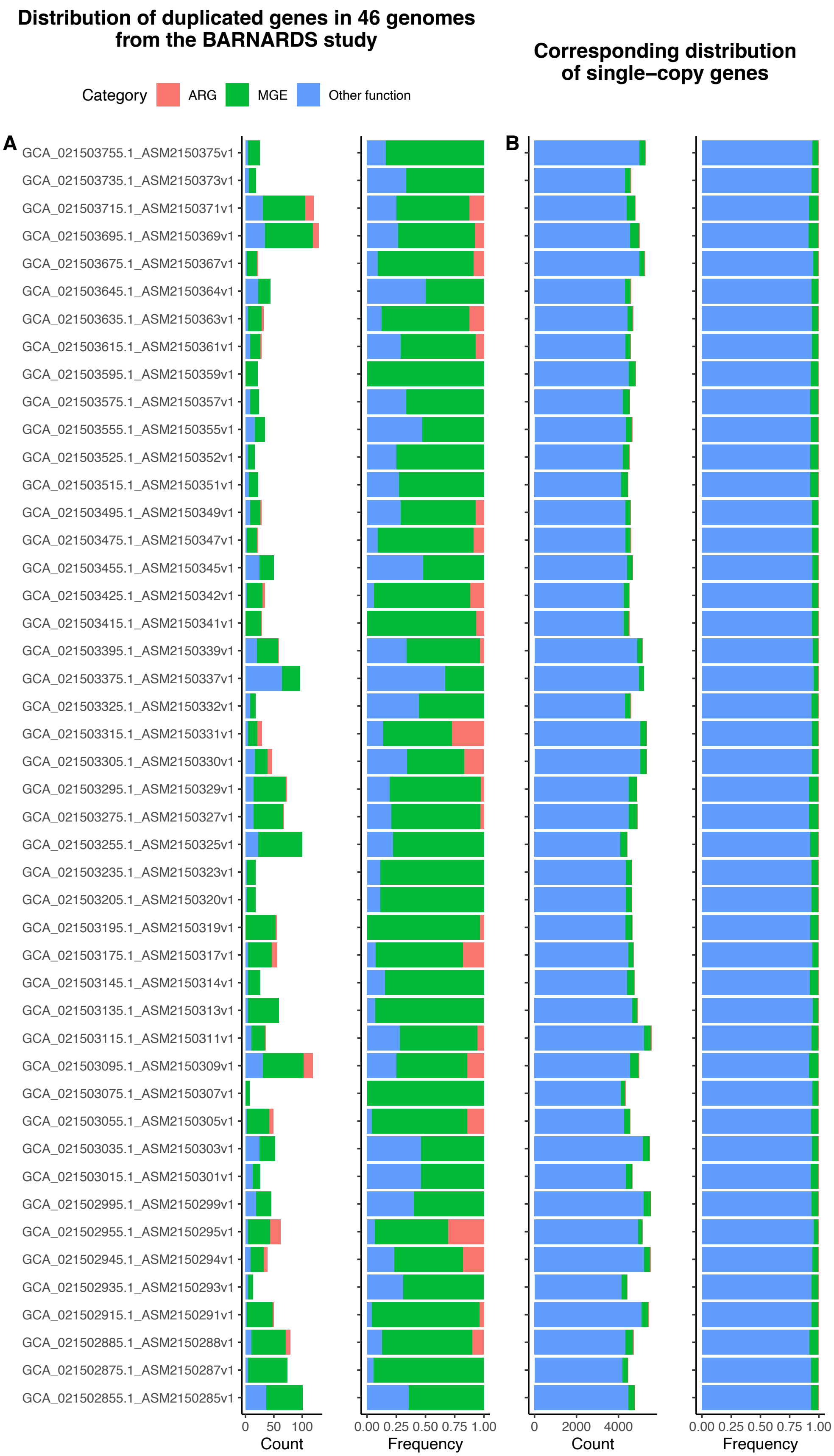

Distribution of duplicated genes in 46 genomes from the BARNARDS study
Corresponding distribution of single−copy genes
Category
ARG
MGE
Other function
A
B
GCA_021503755.1_ASM2150375v1
GCA_021503735.1_ASM2150373v1
GCA_021503715.1_ASM2150371v1
GCA_021503695.1_ASM2150369v1
GCA_021503675.1_ASM2150367v1
GCA_021503645.1_ASM2150364v1
GCA_021503635.1_ASM2150363v1
GCA_021503615.1_ASM2150361v1
GCA_021503595.1_ASM2150359v1
GCA_021503575.1_ASM2150357v1
GCA_021503555.1_ASM2150355v1
GCA_021503525.1_ASM2150352v1
GCA_021503515.1_ASM2150351v1
GCA_021503495.1_ASM2150349v1
GCA_021503475.1_ASM2150347v1
GCA_021503455.1_ASM2150345v1
GCA_021503425.1_ASM2150342v1
GCA_021503415.1_ASM2150341v1
GCA_021503395.1_ASM2150339v1
GCA_021503375.1_ASM2150337v1
GCA_021503325.1_ASM2150332v1
GCA_021503315.1_ASM2150331v1
GCA_021503305.1_ASM2150330v1
GCA_021503295.1_ASM2150329v1
GCA_021503275.1_ASM2150327v1
GCA_021503255.1_ASM2150325v1
GCA_021503235.1_ASM2150323v1
GCA_021503205.1_ASM2150320v1
GCA_021503195.1_ASM2150319v1
GCA_021503175.1_ASM2150317v1
GCA_021503145.1_ASM2150314v1
GCA_021503135.1_ASM2150313v1
GCA_021503115.1_ASM2150311v1
GCA_021503095.1_ASM2150309v1
GCA_021503075.1_ASM2150307v1
GCA_021503055.1_ASM2150305v1
GCA_021503035.1_ASM2150303v1
GCA_021503015.1_ASM2150301v1
GCA_021502995.1_ASM2150299v1
GCA_021502955.1_ASM2150295v1
GCA_021502945.1_ASM2150294v1
GCA_021502935.1_ASM2150293v1
GCA_021502915.1_ASM2150291v1
GCA_021502885.1_ASM2150288v1
GCA_021502875.1_ASM2150287v1
GCA_021502855.1_ASM2150285v1
0 50 100
Count
0.00 0.25 0.50 0.75 1.00
Frequency
0 2000 4000
Count
0.00 0.25 0.50 0.75 1.00
Frequency

Supplementary Figure 8. Distribution of duplicated ARGs in 149 clinical antibiotic resistant isolates sequenced by Mahmud et al. (2022). Hybrid genome assemblies were downloaded from NCBI BioProject PRJNA824420. A) The number of duplicated genes in the 149 isolates is highly variable. 36 out of the 149 isolates harbor duplicated antibiotic resistance genes. B) All 149 isolates have similar numbers and fractions of single-copy genes encoding mobile genetic element functions and antibiotic resistance.

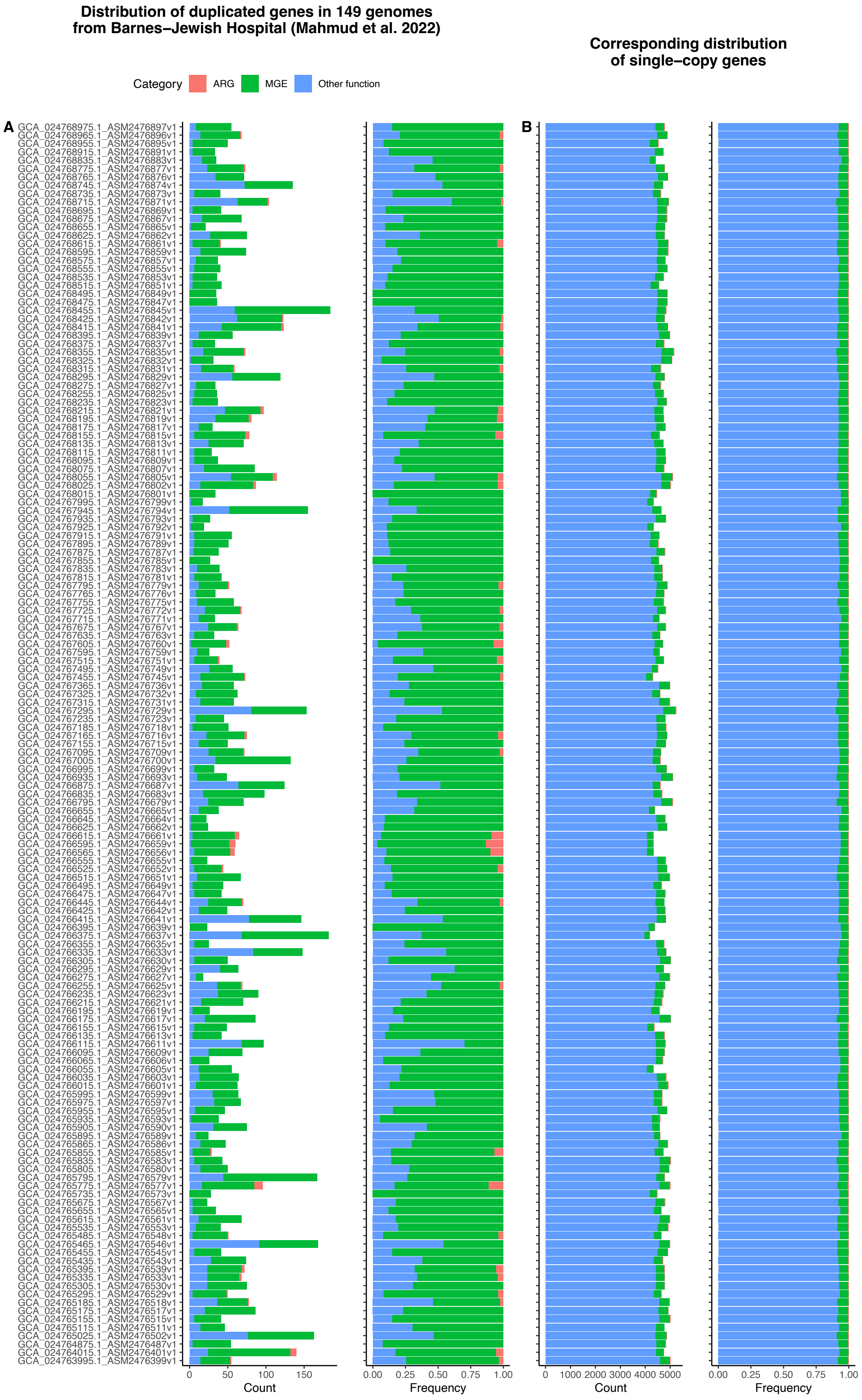
Distribution of duplicated genes in 149 genomes
from Barnes–Jewish Hospital (Mahmud et al. 2022)
Corresponding distribution
of single–copy genes
Category
ARG
MGE
Other function
A
B
GCA_024768975.1_ASM2476897v1
GCA_024768965.1_ASM2476896v1
GCA_024768955.1_ASM2476895v1
GCA_024768915.1_ASM2476891v1
GCA_024768835.1_ASM2476883v1
GCA_024768775.1_ASM2476877v1
GCA_024768765.1_ASM2476876v1
GCA_024768745.1_ASM2476874v1
GCA_024768735.1_ASM2476873v1
GCA_024768715.1_ASM2476871v1
GCA_024768695.1_ASM2476869v1
GCA_024768675.1_ASM2476867v1
GCA_024768655.1_ASM2476865v1
GCA_024768625.1_ASM2476862v1
GCA_024768615.1_ASM2476861v1
GCA_024768595.1_ASM2476859v1
GCA_024768575.1_ASM2476857v1
GCA_024768555.1_ASM2476855v1
GCA_024768535.1_ASM2476853v1
GCA_024768515.1_ASM2476851v1
GCA_024768495.1_ASM2476849v1
GCA_024768475.1_ASM2476847v1
GCA_024768455.1_ASM2476845v1
GCA_024768425.1_ASM2476842v1
GCA_024768415.1_ASM2476841v1
GCA_024768395.1_ASM2476839v1
GCA_024768375.1_ASM2476837v1
GCA_024768355.1_ASM2476835v1
GCA_024768325.1_ASM2476832v1
GCA_024768315.1_ASM2476831v1
GCA_024768295.1_ASM2476829v1
GCA_024768275.1_ASM2476827v1
GCA_024768255.1_ASM2476825v1
GCA_024768235.1_ASM2476823v1
GCA_024768215.1_ASM2476821v1
GCA_024768195.1_ASM2476819v1
GCA_024768175.1_ASM2476817v1
GCA_024768155.1_ASM2476815v1
GCA_024768135.1_ASM2476813v1
GCA_024768115.1_ASM2476811v1
GCA_024768095.1_ASM2476809v1
GCA_024768075.1_ASM2476807v1
GCA_024768055.1_ASM2476805v1
GCA_024768025.1_ASM2476802v1
GCA_024768015.1_ASM2476801v1
GCA_024767995.1_ASM2476799v1
GCA_024767945.1_ASM2476794v1
GCA_024767935.1_ASM2476793v1
GCA_024767925.1_ASM2476792v1
GCA_024767915.1_ASM2476791v1
GCA_024767895.1_ASM2476789v1
GCA_024767875.1_ASM2476787v1
GCA_024767855.1_ASM2476785v1
GCA_024767835.1_ASM2476783v1
GCA_024767815.1_ASM2476781v1
GCA_024767795.1_ASM2476779v1
GCA_024767765.1_ASM2476776v1
GCA_024767755.1_ASM2476775v1
GCA_024767725.1_ASM2476772v1
GCA_024767715.1_ASM2476771v1
GCA_024767675.1_ASM2476767v1
GCA_024767635.1_ASM2476763v1
GCA_024767605.1_ASM2476760v1
GCA_024767595.1_ASM2476759v1
GCA_024767515.1_ASM2476751v1
GCA_024767495.1_ASM2476749v1
GCA_024767455.1_ASM2476745v1
GCA_024767365.1_ASM2476736v1
GCA_024767325.1_ASM2476732v1
GCA_024767315.1_ASM2476731v1
GCA_024767295.1_ASM2476729v1
GCA_024767235.1_ASM2476723v1
GCA_024767185.1_ASM2476718v1
GCA_024767165.1_ASM2476716v1
GCA_024767155.1_ASM2476715v1
GCA_024767095.1_ASM2476709v1
GCA_024767005.1_ASM2476700v1
GCA_024766995.1_ASM2476699v1
GCA_024766935.1_ASM2476693v1
GCA_024766875.1_ASM2476687v1
GCA_024766835.1_ASM2476683v1
GCA_024766795.1_ASM2476679v1
GCA_024766655.1_ASM2476665v1
GCA_024766645.1_ASM2476664v1
GCA_024766625.1_ASM2476662v1
GCA_024766615.1_ASM2476661v1
GCA_024766595.1_ASM2476659v1
GCA_024766565.1_ASM2476656v1
GCA_024766555.1_ASM2476655v1
GCA_024766525.1_ASM2476652v1
GCA_024766515.1_ASM2476651v1
GCA_024766495.1_ASM2476649v1
GCA_024766475.1_ASM2476647v1
GCA_024766445.1_ASM2476644v1
GCA_024766425.1_ASM2476642v1
GCA_024766415.1_ASM2476641v1
GCA_024766395.1_ASM2476639v1
GCA_024766375.1_ASM2476637v1
GCA_024766355.1_ASM2476635v1
GCA_024766335.1_ASM2476633v1
GCA_024766305.1_ASM2476630v1
GCA_024766295.1_ASM2476629v1
GCA_024766275.1_ASM2476627v1
GCA_024766255.1_ASM2476625v1
GCA_024766235.1_ASM2476623v1
GCA_024766215.1_ASM2476621v1
GCA_024766195.1_ASM2476619v1
GCA_024766175.1_ASM2476617v1
GCA_024766155.1_ASM2476615v1
GCA_024766135.1_ASM2476613v1
GCA_024766115.1_ASM2476611v1
GCA_024766095.1_ASM2476609v1
GCA_024766065.1_ASM2476606v1
GCA_024766055.1_ASM2476605v1
GCA_024766035.1_ASM2476603v1
GCA_024766015.1_ASM2476601v1
GCA_024765995.1_ASM2476599v1
GCA_024765975.1_ASM2476597v1
GCA_024765955.1_ASM2476595v1
GCA_024765935.1_ASM2476593v1
GCA_024765905.1_ASM2476590v1
GCA_024765895.1_ASM2476589v1
GCA_024765865.1_ASM2476586v1
GCA_024765855.1_ASM2476585v1
GCA_024765835.1_ASM2476583v1
GCA_024765805.1_ASM2476580v1
GCA_024765795.1_ASM2476579v1
GCA_024765775.1_ASM2476577v1
GCA_024765735.1_ASM2476573v1
GCA_024765675.1_ASM2476567v1
GCA_024765655.1_ASM2476565v1
GCA_024765615.1_ASM2476561v1
GCA_024765535.1_ASM2476553v1
GCA_024765485.1_ASM2476548v1
GCA_024765465.1_ASM2476546v1
GCA_024765455.1_ASM2476545v1
GCA_024765435.1_ASM2476543v1
GCA_024765395.1_ASM2476539v1
GCA_024765335.1_ASM2476533v1
GCA_024765305.1_ASM2476530v1
GCA_024765295.1_ASM2476529v1
GCA_024765185.1_ASM2476518v1
GCA_024765175.1_ASM2476517v1
GCA_024765155.1_ASM2476515v1
GCA_024765115.1_ASM2476511v1
GCA_024765025.1_ASM2476502v1
GCA_024764875.1_ASM2476487v1
GCA_024764015.1_ASM2476401v1
GCA_024763995.1_ASM2476399v1
0
50
100
150
Count
0.00
0.25
0.50
0.75
1.00
Frequency
0
1000
2000
3000
4000
5000
Count
0.00
0.25
0.50
0.75
1.00
Frequency

Supplementary Figure 9. Distribution of duplicated ARGs in 114 clinical antibiotic-resistant isolates from an Australian ICU (Hawkey et al. 2022). Complete genome assemblies were downloaded from NCBI BioProject PRJNA646837. A) The number of duplicated genes in the 114 isolates is highly variable. 20 out of the 114 isolates harbor duplicated antibiotic resistance genes. B) All 114 isolates have similar fractions of single-copy genes encoding mobile genetic element functions and antibiotic resistance.

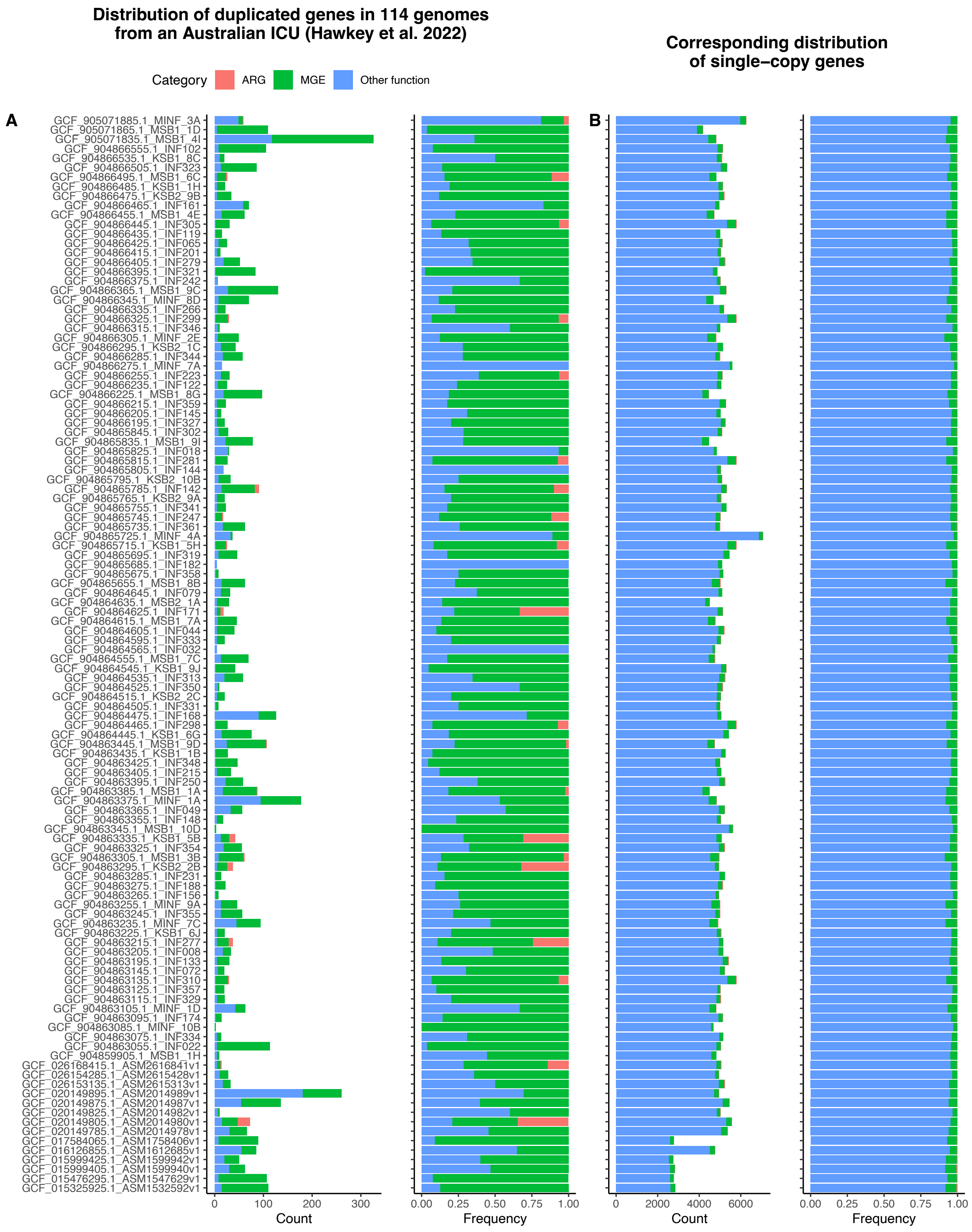

Distribution of duplicated genes in 114 genomes
from an Australian ICU (Hawkey et al. 2022)
Corresponding distribution
of single-copy genes
Category
ARG
MGE
Other function
A
B
GCF_905071885.1_MINF_3A
GCF_905071865.1_MSB1_1D
GCF_905071835.1_MSB1_4I
GCF_904866555.1_INF102
GCF_904866535.1_KSB1_8C
GCF_904866505.1_INF323
GCF_904866495.1_MSB1_6C
GCF_904866485.1_KSB1_1H
GCF_904866475.1_KSB2_9B
GCF_904866465.1_INF161
GCF_904866455.1_MSB1_4E
GCF_904866445.1_INF305
GCF_904866435.1_INF119
GCF_904866425.1_INF065
GCF_904866415.1_INF201
GCF_904866405.1_INF279
GCF_904866395.1_INF321
GCF_904866375.1_INF242
GCF_904866365.1_MSB1_9C
GCF_904866345.1_MINF_8D
GCF_904866335.1_INF266
GCF_904866325.1_INF299
GCF_904866315.1_INF346
GCF_904866305.1_MINF_2E
GCF_904866295.1_KSB2_1C
GCF_904866285.1_INF344
GCF_904866275.1_MINF_7A
GCF_904866255.1_INF223
GCF_904866235.1_INF122
GCF_904866225.1_MSB1_8G
GCF_904866215.1_INF359
GCF_904866205.1_INF145
GCF_904866195.1_INF327
GCF_904865845.1_INF302
GCF_904865835.1_MSB1_9I
GCF_904865825.1_INF018
GCF_904865815.1_INF281
GCF_904865805.1_INF144
GCF_904865795.1_KSB2_10B
GCF_904865785.1_INF142
GCF_904865765.1_KSB2_9A
GCF_904865755.1_INF341
GCF_904865745.1_INF247
GCF_904865735.1_INF361
GCF_904865725.1_MINF_4A
GCF_904865715.1_KSB1_5H
GCF_904865695.1_INF319
GCF_904865685.1_INF182
GCF_904865675.1_INF358
GCF_904865655.1_MSB1_8B
GCF_904864645.1_INF079
GCF_904864635.1_MSB2_1A
GCF_904864625.1_INF171
GCF_904864615.1_MSB1_7A
GCF_904864605.1_INF044
GCF_904864595.1_INF333
GCF_904864565.1_INF032
GCF_904864555.1_MSB1_7C
GCF_904864545.1_KSB1_9J
GCF_904864535.1_INF313
GCF_904864525.1_INF350
GCF_904864515.1_KSB2_2C
GCF_904864505.1_INF331
GCF_904864475.1_INF168
GCF_904864465.1_INF298
GCF_904864445.1_KSB1_6G
GCF_904863445.1_MSB1_9D
GCF_904863435.1_KSB1_1B
GCF_904863425.1_INF348
GCF_904863405.1_INF215
GCF_904863395.1_INF250
GCF_904863385.1_MSB1_1A
GCF_904863375.1_MINF_1A
GCF_904863365.1_INF049
GCF_904863355.1_INF148
GCF_904863345.1_MSB1_10D
GCF_904863335.1_KSB1_5B
GCF_904863325.1_INF354
GCF_904863305.1_MSB1_3B
GCF_904863295.1_KSB2_2B
GCF_904863285.1_INF231
GCF_904863275.1_INF188
GCF_904863265.1_INF156
GCF_904863255.1_MINF_9A
GCF_904863245.1_INF355
GCF_904863235.1_MINF_7C
GCF_904863225.1_KSB1_6J
GCF_904863215.1_INF277
GCF_904863205.1_INF008
GCF_904863195.1_INF133
GCF_904863145.1_INF072
GCF_904863135.1_INF310
GCF_904863125.1_INF357
GCF_904863115.1_INF329
GCF_904863105.1_MINF_1D
GCF_904863095.1_INF174
GCF_904863085.1_MINF_10B
GCF_904863075.1_INF334
GCF_904863055.1_INF022
GCF_904859905.1_MSB1_1H
GCF_026168415.1_ASM2616841v1
GCF_026154285.1_ASM2615428v1
GCF_026153135.1_ASM2615313v1
GCF_020149895.1_ASM2014989v1
GCF_020149875.1_ASM2014987v1
GCF_020149825.1_ASM2014982v1
GCF_020149805.1_ASM2014980v1
GCF_020149785.1_ASM2014978v1
GCF_017584065.1_ASM1758406v1
GCF_016126855.1_ASM1612685v1
GCF_015999425.1_ASM1599942v1
GCF_015999405.1_ASM1599940v1
GCF_015476295.1_ASM1547629v1
GCF_015325925.1_ASM1532592v1
0
100
200
300
Count
0.00
0.25
0.50
0.75
1.00
Frequency
0
2000
4000
6000
Count
0.00
0.25
0.50
0.75
1.00
Frequency

Supplementary Figure 10. Plasmid copy number relative to chromosome among 114 clinical antibiotic-resistant isolates from an Australian ICU. Complete genome assemblies were downloaded from NCBI BioProject PRJNA646837. Plasmid copy number relative to chromosome was measured by dividing the average Illumina short-read sequencing coverage per plasmid by the average Illumina short-read sequencing coverage per chromosome in each genome assembly.

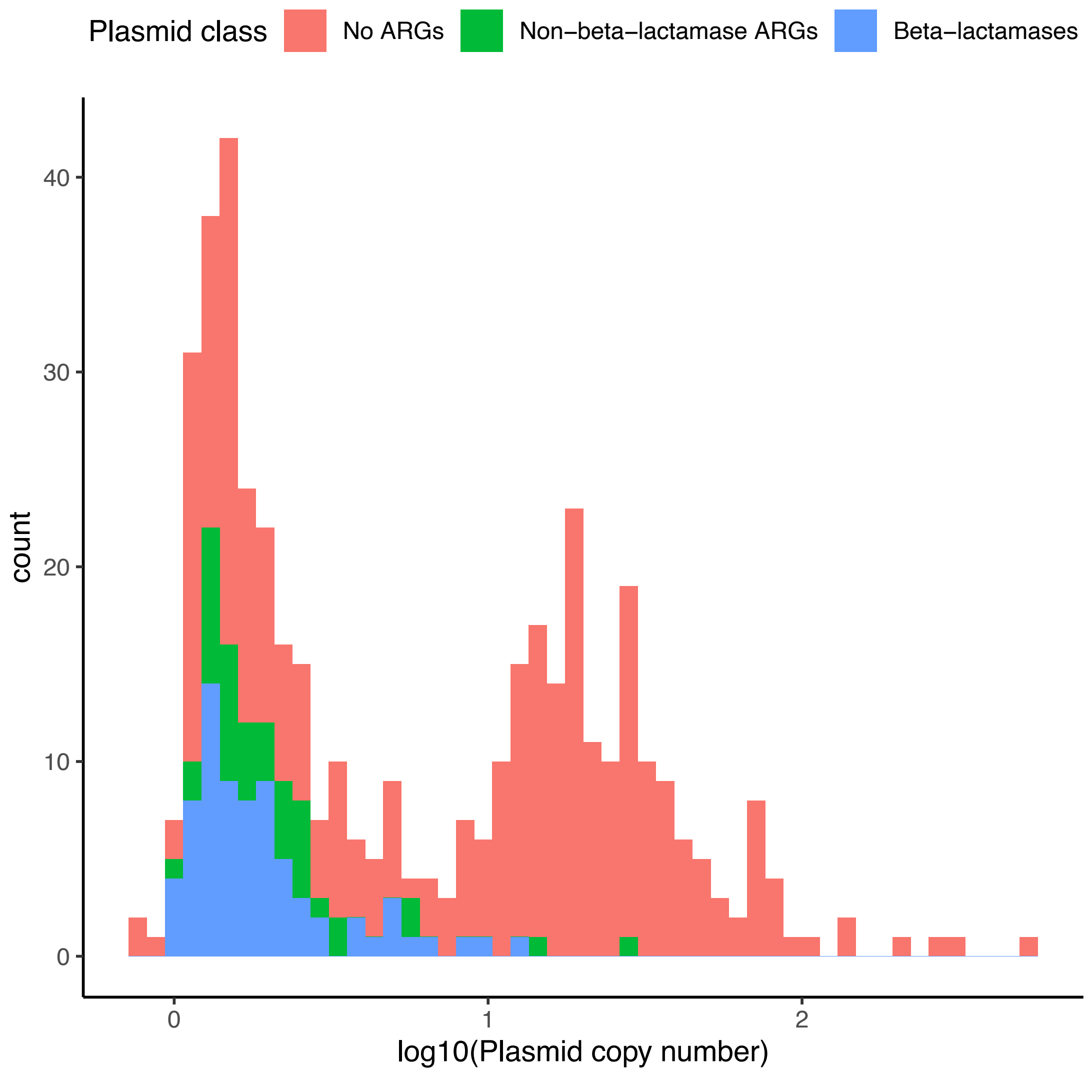

Supplementary Figure 11. Genomic distribution of duplicated genes (D-genes). D-genes encoded solely on plasmids are more likely to encode functions other than those associated with mobile genetic elements, in comparison to both D-genes encoded solely on the chromosome, and D-genes encoded on plasmids and the chromosome.

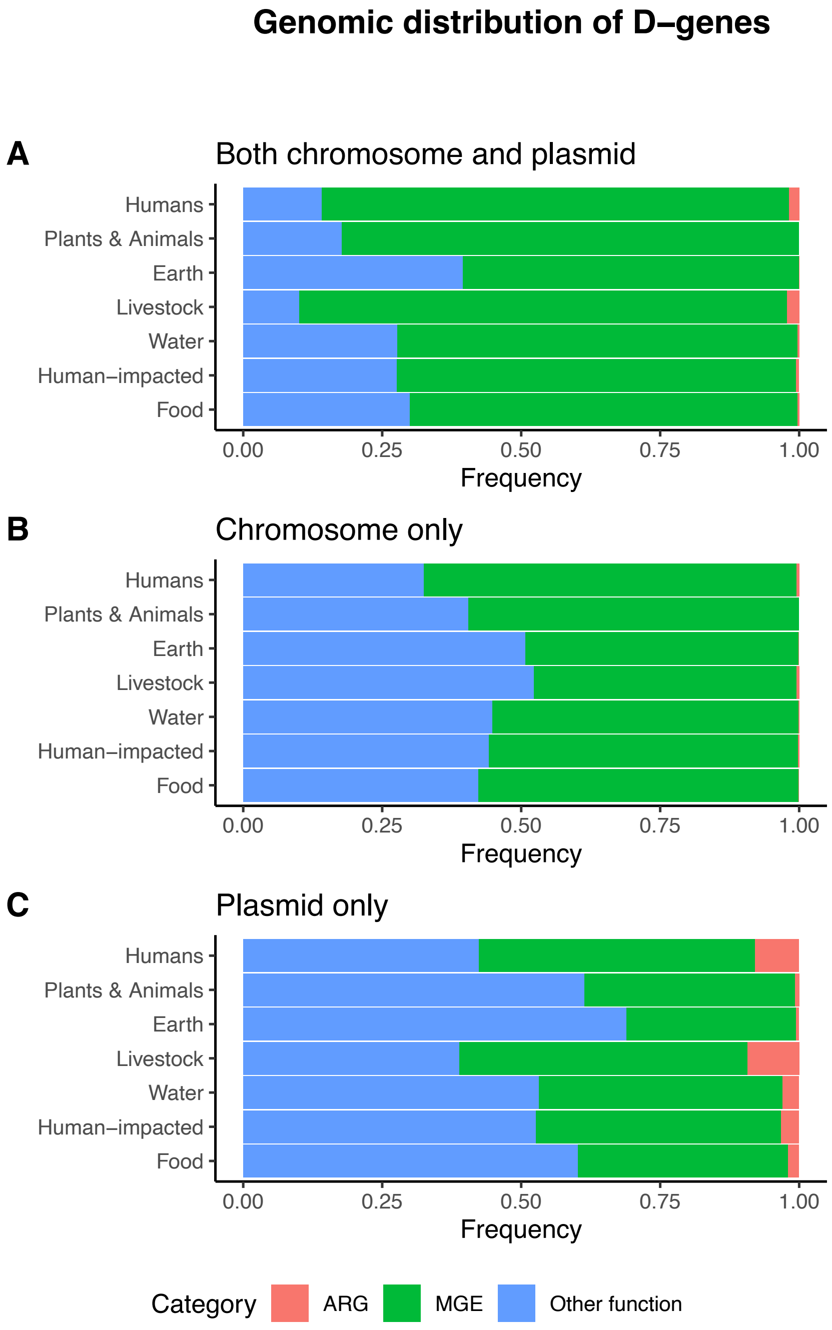

Supplementary Figure 12. Non-random evolutionary forces such as natural selection and horizontal gene transfer drive the ecological distributions of duplicated ARGs and MGE-associated genes away from equilibrium. Suppose no evolutionary forces such as selection, horizontal gene transfer, or associations with mobile genetic elements affect the probability that a gene undergoes gene duplication. In this scenario, the probability that a gene of a given functional class is duplicated should be proportional to the number of single-copy genes of that functional class in the ecological category. Deviations from this null expectation indicates that the frequency of duplicated genes is being driven away from this equilibrium by evolutionary forces, such as selection and HGT.

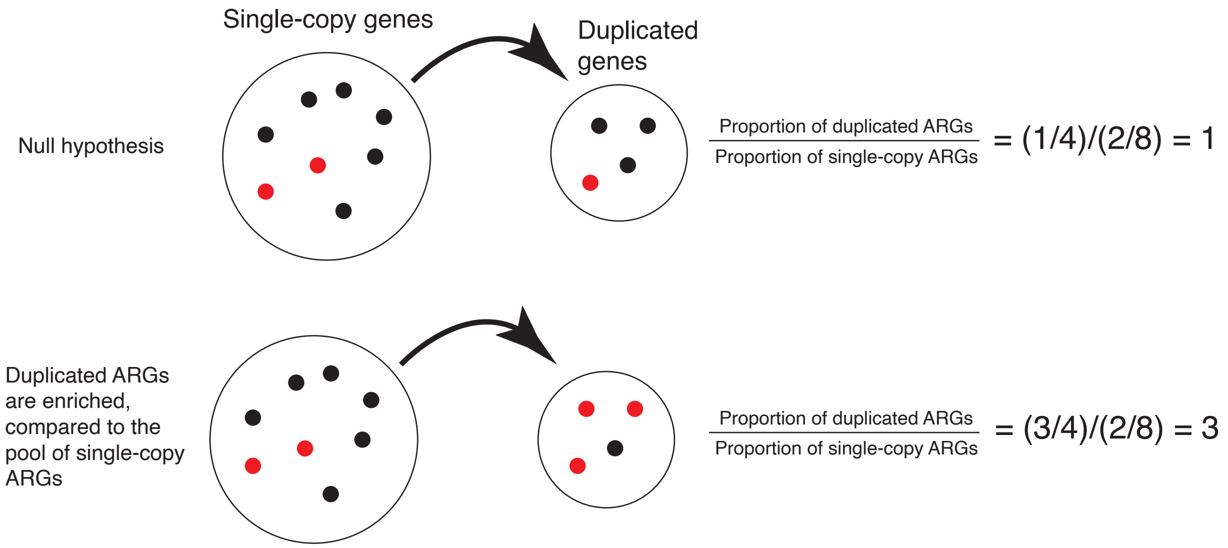

Supplementary Figure 13. Full distribution of transposases associated with ARGs in regions of consecutive duplicated genes.

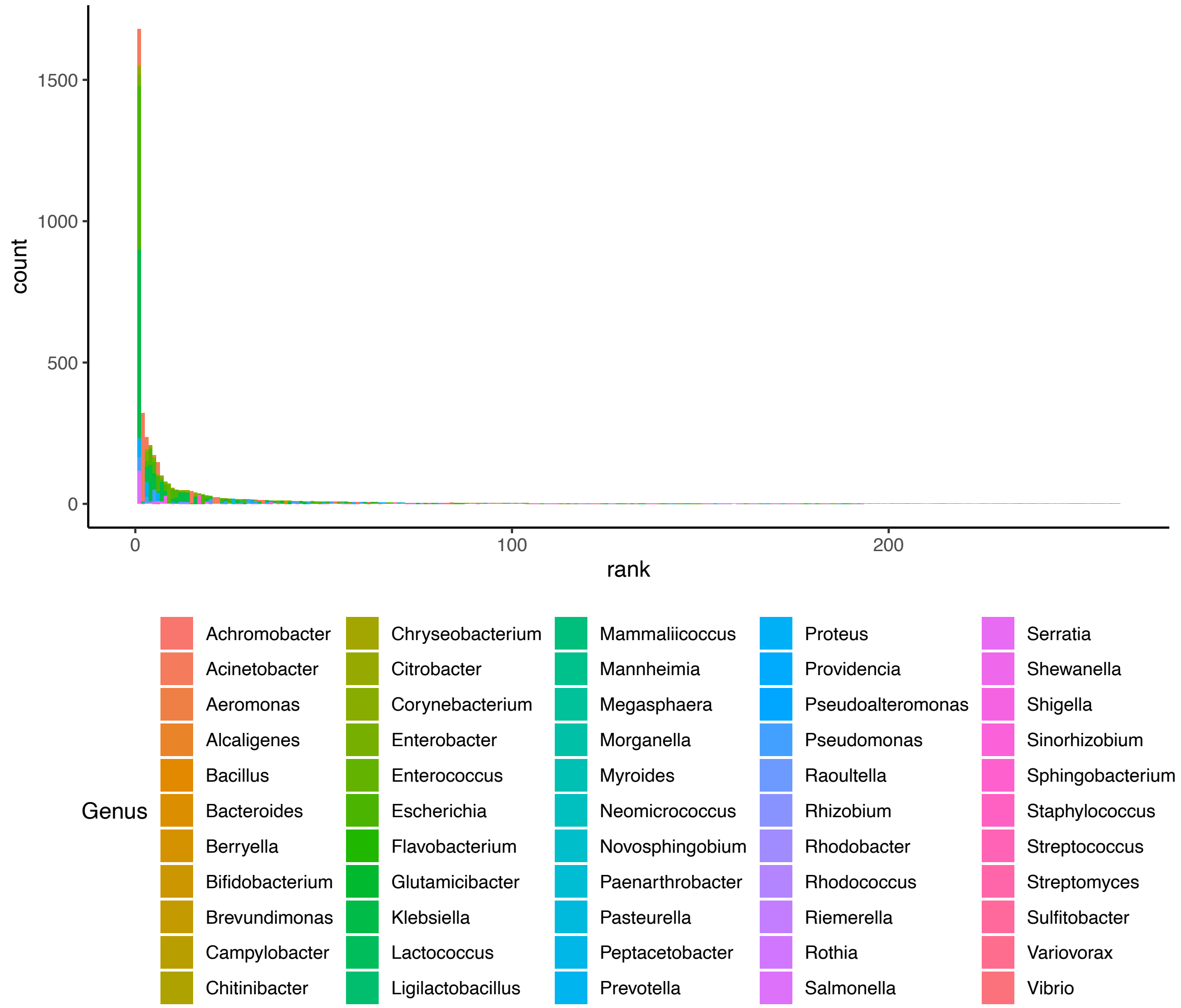

Supplementary Figure 14. Bacteria isolated from humans and livestock are much more likely to have duplicated antibiotic resistance genes (D-ARGs) compared to bacteria isolated from other environments; furthermore, D-ARGs are enriched on the chromosomes and plasmids of bacteria isolated from humans and livestock (using CARD to annotate ARGs and mobileOG-db to annotate MGE-associated genes). Error bars are 95% binomial proportion confidence intervals, calculated using the formula $p \pm Z_{\alpha/2}\sqrt{(\frac{p(1-p)}{n})}$, where *p* is the proportion, *n* is the sample size, and $Z_{\alpha/2}$ = 1.96.

A. D-ARGs are specifically enriched in bacterial isolates from humans and livestock.

B. The vast majority of isolates contain at least one single-copy antibiotic resistance gene (S-ARG).

C. The vast majority of isolates contain at least one duplicated gene (D-gene).

D. D-ARGs represent a higher fraction of genes found in bacteria isolated from humans and livestock compared to bacteria in the other ecological categories.

E. Chromosomal D-ARGs are enriched in bacteria isolated from humans and livestock.

F. Plasmid D-ARGs are enriched in bacteria isolated from humans and livestock.

G. S-ARGs represent a higher fraction of genes found in bacteria isolated from humans and livestock compared to the other ecological categories.

H. Chromosomal S-ARGs are enriched in humans and livestock.

I. Plasmid S-ARGs are enriched in bacteria isolated from humans and livestock.

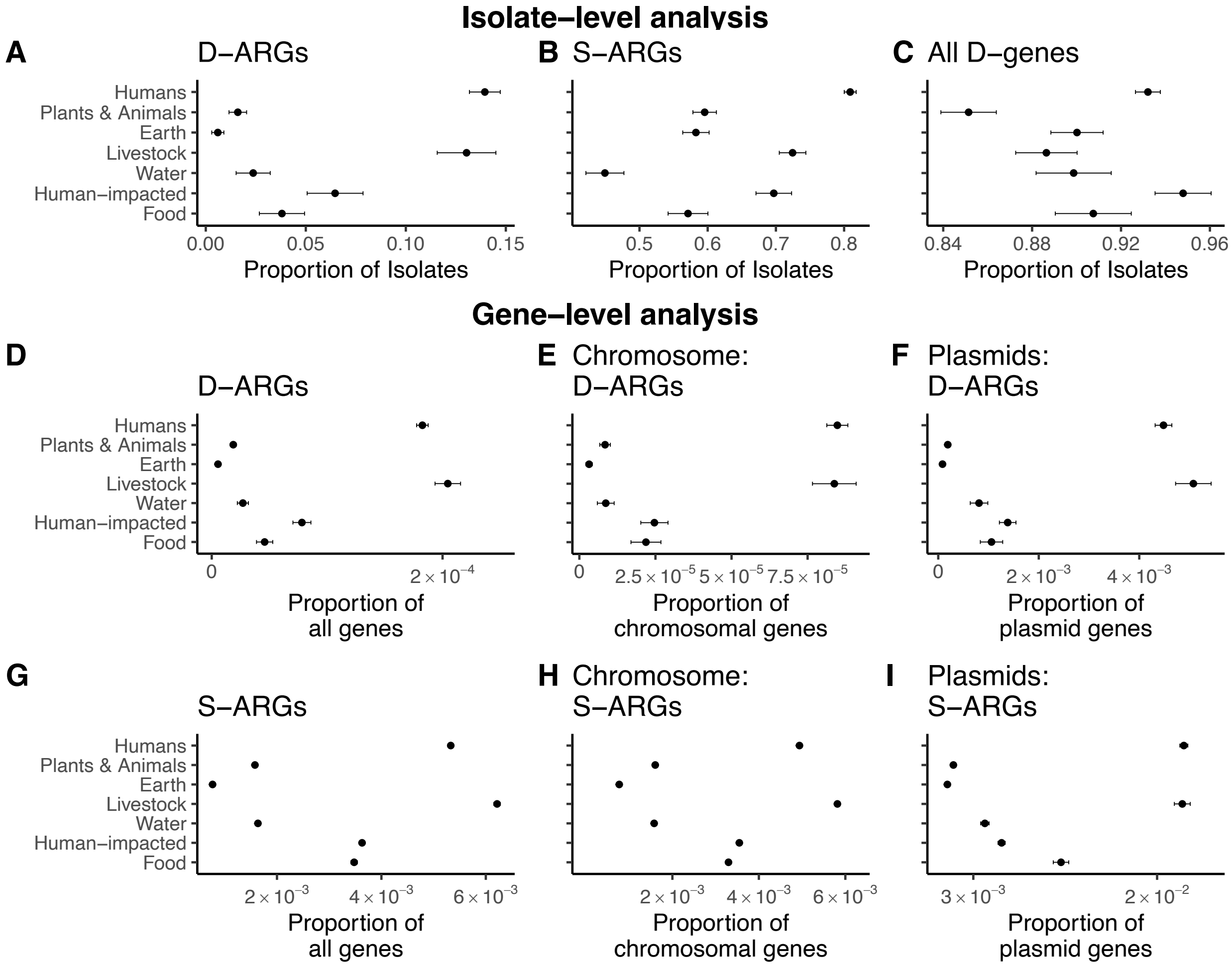

Isolate–level analysis
A
D–ARGs
B S–ARGs
C All D–genes
Humans
Plants & Animals
Earth
Livestock
Water
Human–impacted
Food
Proportion of Isolates
Gene–level analysis
D
D–ARGs
E Chromosome: D–ARGs
F Plasmids: D–ARGs
Proportion of all genes
Proportion of chromosomal genes
Proportion of plasmid genes
G
S–ARGs
H Chromosome: S–ARGs
I Plasmids: S–ARGs

Supplementary Figure 15. D-ARGs are enriched on the chromosomes and plasmids of bacteria isolated from humans and livestock (using CARD to annotate ARGs and mobileOG-db to annotate MGE-associated genes). Error bars are 95% binomial proportion confidence intervals, calculated using the formula $p \pm Z_{\alpha/2}\sqrt(\frac{p(1-p)}{n})$, where *p* is the proportion, *n* is the sample size, and $Z_{\alpha/2}$ = 1.96.

A. Bacterial isolates with D-ARGs on chromosomes are specifically associated with humans and livestock.

B. Bacterial isolates with D-ARGs on plasmids are specifically associated with humans and livestock.

C. Most isolates contain at least one single-copy ARG on their chromosome.

D. Bacterial isolates with S-ARGs on plasmids are specifically associated with humans, livestock, and human-impacted environments.

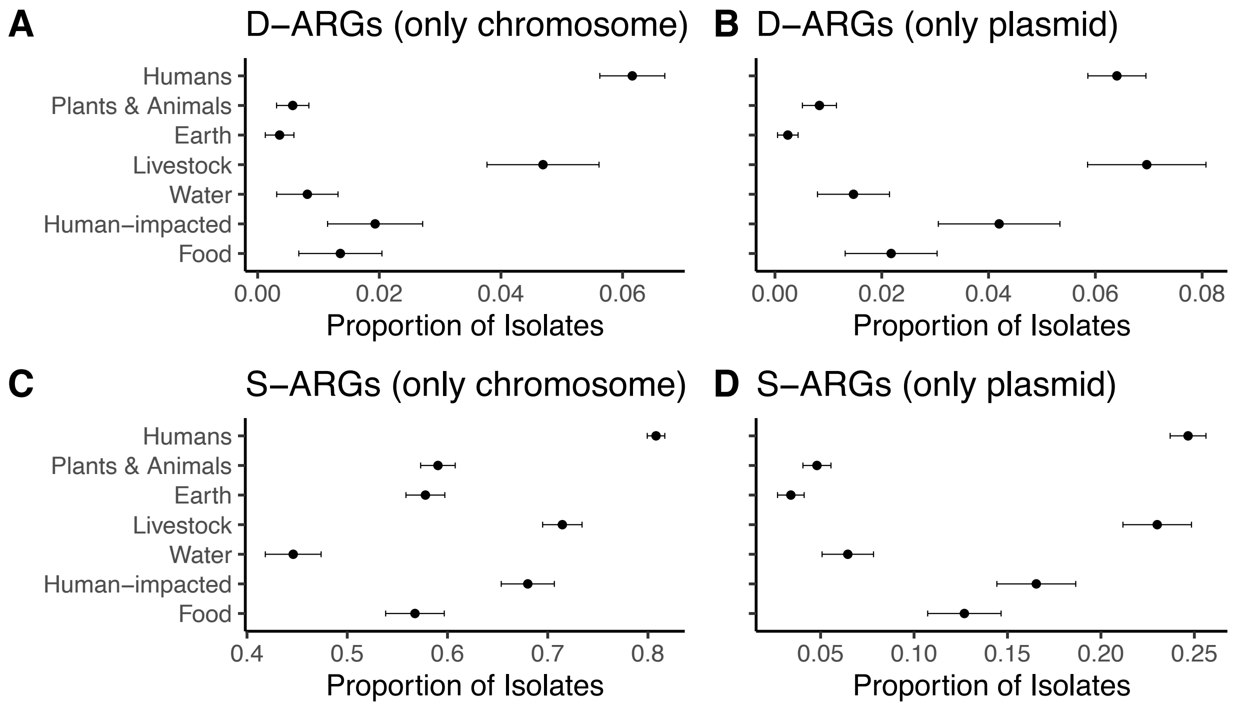

Supplementary Figure 16. Selection, horizontal gene transfer, and mobile genetic elements shape the ecological distribution of duplicated genes (using CARD to annotate ARGs and mobileOG-db to annotate MGE-associated genes). Proteins associated with mobile genetic elements (MGEs) are shown in green; proteins encoded by antibiotic resistance genes (ARGs) are in red; and all other proteins are shown in blue.

A. Across all ecological categories, ~50% duplicated genes (D-genes) on chromosomes and plasmids are associated with MGEs.

B. MGE-associated proteins account for <10% of single-copy genes (S-genes) on chromosomes, and 5-25% of S-genes on plasmids.

C. Duplicated ARGs (D-ARGs) are enriched in humans, livestock, water, and human-impacted environments, and are depleted in isolates from plants and animals, while duplicated genes associated with mobile genetic element functions are enriched in all ecological categories. The red dashed line indicates the null hypothesis.

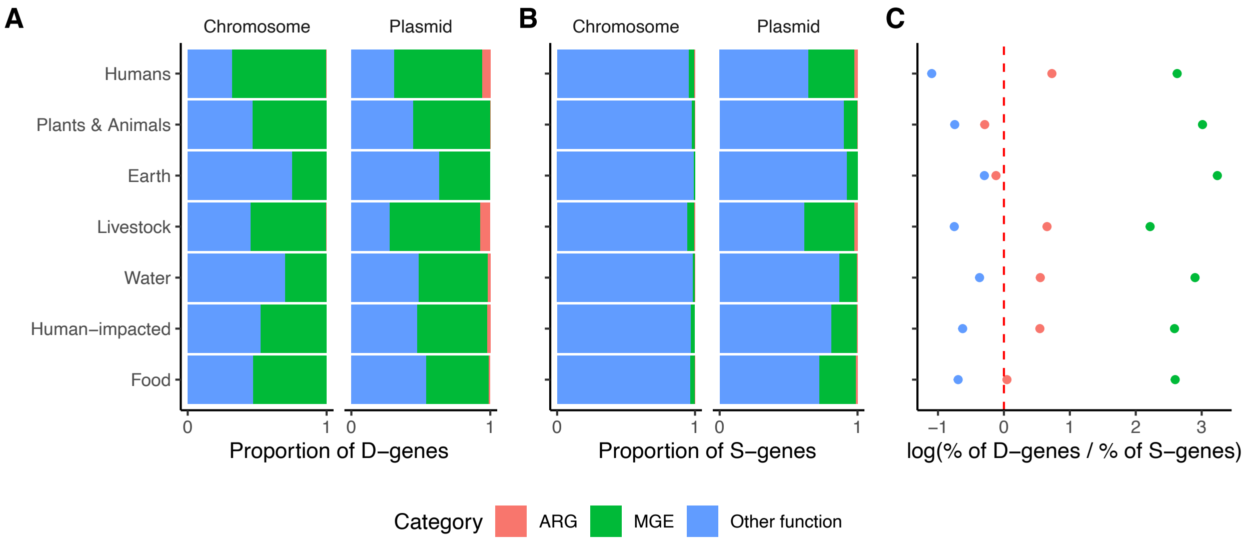

Supplementary Data Files

File Name: Supplementary Data 1

Description: Pluto computational notebook, written in the Julia programming language, allowing for user interaction with the deterministic mathematical model.

File Name: Supplementary Data 2

Description: Mutations found in the evolution experiments.

File Name: Supplementary Data 3

Description: Ecologically annotated complete genomes, annotated by presence and absence of duplicated ARGs.

File Name: Supplementary Data 4

Description: Duplicated ARGs found in the Complete Genomes from NCBI RefSeq.

File Name: Supplementary Data 5

Description: MOBTyper annotations of plasmid found in the Complete Genomes from NCBI RefSeq.

File Name: Source Data

Description: Source Data for main figures.
