# Supplementary material for: Duplicated antibiotic resistance genes reveal ongoing selection and horizontal gene transfer in bacteria
Source: Nat Commun. 2024 Feb 16;15:1449. doi: 10.1038/s41467-024-45638-9 (PMC10873360; doi:10.1038/s41467-024-45638-9)
Supplement: Supplementary file 3 — Description of Additional Supplementary Files [file 41467_2024_45638_MOESM3_ESM.pdf]

File Name: Supplementary Data 1
Description: Pluto computational notebook, written in the Julia programming language, allowing for user interaction with the deterministic mathematical model.

File Name: Supplementary Data 2
Description: Mutations found in the evolution experiments.

File Name: Supplementary Data 3
Description: Ecologically annotated complete genomes, annotated by presence and absence of duplicated ARGs.

File Name: Supplementary Data 4
Description: Duplicated ARGs found in the Complete Genomes from NCBI RefSeq.

File Name: Supplementary Data 5
Description: MOBTyper annotations of plasmid found in the Complete Genomes from NCBI RefSeq.
