# Supplementary material for: Duplicated antibiotic resistance genes reveal ongoing selection and horizontal gene transfer in bacteria
Source: Nat Commun. 2024 Feb 16;15:1449. doi: 10.1038/s41467-024-45638-9 (PMC10873360; doi:10.1038/s41467-024-45638-9)
Supplement: Supplementary file 9 — Reporting Summary [file 41467_2024_45638_MOESM9_ESM.pdf]

nature portfolio

Corresponding author(s): Lingchong You

Last updated by author(s): Dec 26, 2023

Reporting Summary

Nature Portfolio wishes to improve the reproducibility of the work that we publish. This form provides structure for consistency and transparency in reporting. For further information on Nature Portfolio policies, see our Editorial Policies and the Editorial Policy Checklist.

Statistics

For all statistical analyses, confirm that the following items are present in the figure legend, table legend, main text, or Methods section.

n/a	Confirmed	
☐	☒	The exact sample size (*n*) for each experimental group/condition, given as a discrete number and unit of measurement
☐	☒	A statement on whether measurements were taken from distinct samples or whether the same sample was measured repeatedly
☐	☒	The statistical test(s) used AND whether they are one- or two-sided<br>*Only common tests should be described solely by name; describe more complex techniques in the Methods section.*
☐	☒	A description of all covariates tested
☐	☒	A description of any assumptions or corrections, such as tests of normality and adjustment for multiple comparisons
☐	☒	A full description of the statistical parameters including central tendency (e.g. means) or other basic estimates (e.g. regression coefficient) AND variation (e.g. standard deviation) or associated estimates of uncertainty (e.g. confidence intervals)
☐	☒	For null hypothesis testing, the test statistic (e.g. *F*, *t*, *r*) with confidence intervals, effect sizes, degrees of freedom and *P* value noted<br>*Give P values as exact values whenever suitable.*
☐	☒	For Bayesian analysis, information on the choice of priors and Markov chain Monte Carlo settings
☐	☒	For hierarchical and complex designs, identification of the appropriate level for tests and full reporting of outcomes
☒	☐	Estimates of effect sizes (e.g. Cohen's *d*, Pearson's *r*), indicating how they were calculated

Our web collection on statistics for biologists contains articles on many of the points above.

Software and code

Policy information about availability of computer code

Data collection	Python v3.6+, Mash v2.3, Assembly Dereplicator 0.3.1, NCBI sra-toolkit 3.0.5, DIAMOND 2.1.6, kallisto 0.48.0
Data analysis	Python v3.6+, R v4.0+, Julia v1.8+, breseq v0.37+. In addition, computer code (and instructions for how to run) are available at: https://github.com/youlab/youlab-ARG-duplications and https://github.com/rohanmaddamsetti/AR-gene-plasmid-analysis.

For manuscripts utilizing custom algorithms or software that are central to the research but not yet described in published literature, software must be made available to editors and reviewers. We strongly encourage code deposition in a community repository (e.g. GitHub). See the Nature Portfolio guidelines for submitting code & software for further information.

Data

Policy information about availability of data

All manuscripts must include a data availability statement. This statement should provide the following information, where applicable:

- Accession codes, unique identifiers, or web links for publicly available datasets
- A description of any restrictions on data availability
- For clinical datasets or third party data, please ensure that the statement adheres to our policy

Source data are provided with this paper. Accessions for the 18,938 complete bacterial genomes from NCBI RefSeq analyzed in this work are listed in Supplementary Data 3. Accessions for the 321 antibiotic-resistant clinical-isolate bacterial complete genomes from NCBI BioProjects PRJNA290784, PRJNA767644, PRJNA824420,

and PRJNA646837 analyzed in this work are listed in the figure legends of Extended Data Figures 6, 7, 8, and 9. A minimum dataset necessary to interpret, verify and extend the research in this article is available from Zenodo (DOI: 10.5281/zenodo.10431250)

Research involving human participants, their data, or biological material

Policy information about studies with human participants or human data. See also policy information about sex, gender (identity/presentation), and sexual orientation and race, ethnicity and racism.

Reporting on sex and gender	N/A
Reporting on race, ethnicity, or other socially relevant groupings	N/A
Population characteristics	N/A
Recruitment	N/A
Ethics oversight	N/A

Note that full information on the approval of the study protocol must also be provided in the manuscript.

Field-specific reporting

Please select the one below that is the best fit for your research. If you are not sure, read the appropriate sections before making your selection.

[x] Life sciences [ ] Behavioural & social sciences [ ] Ecological, evolutionary & environmental sciences

For a reference copy of the document with all sections, see nature.com/documents/nr-reporting-summary-flat.pdf

Life sciences study design

All studies must disclose on these points even when the disclosure is negative.

Sample size	All complete genomes from NCBI RefSeq were analyzed (18,938 genomes after quality controls). In the main evolution experiments, a minimum of 5 biological replicate populations per treatment were evolved. In the additional evolution experiment requested by referees during revision, 10 biological replicate popuations were evolved, with 2 biological replicates per block (antibiotic class). These sample sizes provide sufficient statistical power to detect positive selection by examining parallel evolution across biological replicate populations.
Data exclusions	Genomes were excluded if they failed QC for inclusion into NCBI RefSeq, or the additional QC procedures described in "Methods: Curation of complete bacterial genomes."
Replication	Bioinformatic results were replicated on 4 independent sets of antibiotic-resistant clinical isolate genomes from NCBI BioProjects PRJNA290784, PRJNA767644, PRJNA824420, and PRJNA646837. The main experimental evolution results were replicated using two E. coli strains (DH5a and K12) and 5 antibiotic treatments across 3 different evolution experiments: 1) DH5a + Tetracycline 2) K12 + Tetracycline 3) K12 + Spectinomycin, Kanamycin, Chloramphenicol, and Carbenicillin.
Randomization	Allocation was random.
Blinding	N/A (mathematical modeling + experimental microbiology + bioinformatics study)

Reporting for specific materials, systems and methods

We require information from authors about some types of materials, experimental systems and methods used in many studies. Here, indicate whether each material, system or method listed is relevant to your study. If you are not sure if a list item applies to your research, read the appropriate section before selecting a response.

Materials & experimental systems

n/a	Involved in the study	
[x]	[ ]	Antibodies
[x]	[ ]	Eukaryotic cell lines
[x]	[ ]	Palaeontology and archaeology
[x]	[ ]	Animals and other organisms
[x]	[ ]	Clinical data
[x]	[ ]	Dual use research of concern
[x]	[ ]	Plants

Methods

n/a	Involved in the study	
[x]	[ ]	ChIP-seq
[x]	[ ]	Flow cytometry
[x]	[ ]	MRI-based neuroimaging

Plants

Seed stocks	N/A
Novel plant genotypes	N/A
Authentication	N/A
